# An Aliasing Measure of Factor Effects in Three-Level Regular Designs

**DOI:** 10.3390/e27070680

**Published:** 2025-06-26

**Authors:** Qiuying Chen, Zhiming Li, Zhi Li

**Affiliations:** College of Mathematics and System Science, Xinjiang University, Urumqi 830017, China; 107552300624@stu.xju.edu.cn (Q.C.); lizhi@xju.edu.cn (Z.L.)

**Keywords:** regular design, optimal criterion, aliasing information, algorithm

## Abstract

For three-level regular designs, the confounding from the perspectives of both factor and component effects leads to different results. The aliasing properties of factor effects are more significant than the latter in the experimental model. In this paper, a new three-level aliasing pattern is proposed to evaluate the degree of aliasing among different factors. Based on the classification pattern, a new criterion is introduced for choosing optimal three-level regular designs. Then, we analyze the relationship between the criterion and the existing criteria, including general minimum lower-order confounding, entropy, minimum aberration, and clear effects. The results show that the classification patterns of other criteria can be expressed as functions of our proposed pattern. Further, an aliasing algorithm is provided, and all 27-run, some of the 81-run, and 243-run three-level designs are listed in tables and compared with the rankings under other criteria. A real example is provided to illustrate the proposed methods.

## 1. Introduction

Regular designs are widely applied in agriculture, industry, and other fields. The selection of optimal regular designs for improving yield and shortening testing cycles has garnered attention in theoretical and practical fields. Several criteria have been established for the selection of optimal designs. The first criterion is the maximum resolution (MR) criterion introduced by Box and Hunter [1], which selects designs with higher resolutions. The second one, proposed by Fries and Hunter [2], is the minimum aberration (MA) criterion, which is based on the word-length pattern (WLP). The third criterion, based on the definition of clear effects, was proposed by Wu and Chen [3], which selects designs with the maximum number of clear main effects and two-factor interactions (2fis) [4]. More details of the above criteria can be found in Mukerjee and Wu [5] and Wu and Hamada [6]. Zhang and Wang [7] proved the entropy optimality of orthogonal designs. The characterization of confounding information under these criteria varies, leading to the selection of different optimal designs.

To reveal the essence of the aforementioned criteria, Zhang et al. [8] introduced an aliased effect-number pattern (AENP) for two-level designs. Based on AENP, a general minimum lower-order confounding (GMC) criterion is proposed to evaluate two-level regular designs. Zhang and Mukerjee [9] studied the properties of *s*-level GMC designs via complementary sets. Li et al. [10] introduced the aliased component-number pattern (ACNP) and proposed a three-level GMC criterion. Li et al. [11] proved that classification patterns for MR, MA, and CE can be represented by the ACNP. In this sense, the GMC criterion is more informative and elaborate than other criteria. Up to now, there have been many achievements for three-level GMC designs [12,13]. However, the classification patterns of all the above results are based on the component properties of designs. Unlike the two-level case, the interpretability of component confounding is limited in higher-level designs. It is not easy to distinguish between factor aliasing effectively. Furthermore, the number of components increases exponentially, significantly raising computational complexity. The nonlinear relationships between component effects may introduce unnecessary complexity in modeling [6].

In a practical model, the aliasing between factors with three levels is often more critical to optimize experimental conditions (Jaynes et al. [14], Suriyaamporn et al. [15], and Nieweś [16]). Guo et al. [17] and Cheng et al. [18] screened the optimal process conditions through three-level orthogonal designs and validated the selected results using the entropy weight method. So far, the study of higher-level factor aliasing remains unfulfilled for regular designs with three levels. It is difficult for the existing results to express the aliasing among factor effects. Motivated by the analysis above, we focus on studying three-level factor aliasing. From a factor-based perspective, the aliasing measure aims to reduce computational complexity, make the results easier to interpret, and enhance the practical value for modeling applications. Thus, the main contributions and innovations of our work focus on the following aspects:

(i) A factor aliasing pattern is first proposed to describe the aliasing information of three-level regular designs, and a new criterion is introduced based on the pattern. For the two-level design, the pattern is equivalent to the AENP. However, it differs from the ACNP of three-level designs because of the relationship between factor and component effects.

(ii) We analyze the relationship between the proposed and existing patterns to reveal the essential characteristics of various confounding and alias relations.

(iii) Based on the proposed criterion, we propose an aliasing algorithm of lower-order factors to choose optimal designs. Compared with other criteria, tables list all 27-, some 81-, and 243-run optimal designs.

The main structure of this paper is organized as follows. Section 2 introduces a new measure for describing factor aliasing, known as the aliased factor-number pattern (AFNP) for three-level regular designs, along with a criterion for general minimizing lower-order aliasing based on factor effect (GMAF), which utilizes the AFNP. The relationship between the new criterion and the GMC, entropy, MA, and CE criteria is discussed in Section 3. Section 4 presents an aliasing algorithm of the GMAF criteria and a catalog of the optimal designs for all 27- and some 81- and 243-run designs of resolution IV or higher. Section 5 provides an example to illustrate the application of the new criterion. Section 6 offers a brief conclusion.

## 2. Some Basic Concepts

We first review some basic concepts of three-level designs. Let *n* and *m* be two positive integers with n>m. Define q=n−m and $N=3^{q}$. The numbers 1,2,…,q correspond to *q* independent columns. A $3^{\left(N-1)/2-((N-1)/2-q\right)}$ saturated design $H_{q}$ is determined by the following recursive process:

$$H_{1}=\left\{1\right\},\;H_{r}=\left\{H_{r-1},r,rH_{r-1},r^{2}H_{r-1}\right\},\;r=2,\;\ldots ,q,$$

where $r^{i}H_{r-1}=\left\{r^{i}d:d\in H_{r-1}\right\}$ for r=2,…,q and i=1,2. Here, the operation rd is the element-wise addition modulo 3 over the Galois field of order 3, denoted as GF(3). Thus, $H_{q}=\left\{H_{q-1},q,qH_{q-1},q^{2}H_{q-1}\right\}.$ A $3^{n-m}$ design with *n* factors $d_{1},\;\ldots ,d_{n}$, each having three levels, is constructed by *q* independent columns 1,…,q and *m* additional columns generated from the three-level saturated design $H_{q}$. We call $d_{l_{1}}\times d_{l_{2}}\times \ldots \times d_{li}\left(1\leq i,l_{i}\leq n\right)$ an *i*-order factor interaction (*i*fi) of the design, corresponding to $2^{i-1}$ orthogonal factor-interaction components (fics). In particular, $d_{i}$ is the main effect, and $d_{l_{1}}\times d_{l_{2}}$ means a 2fi.

Consider a $3^{4-1}$ design D={1,2,3,123} with the defining relation $I=1234^{2}$. Suppose that third- and higher-order interactions are negligible. By multiplying both sides of $I=1234^{2}$ by main effect and 2fic, we have $12=34^{2},13=24^{2}$ and $23=14^{2}.$ It is evident that no main effects are aliased with other main effects or 2fics, and six 2fics are confounded with one other 2fic. Suppose any component of a factor effect is confounded with a component of another factor. In that case, the factor is aliased with the corresponding factor, and the symbol “≈” denotes that two factors are aliased. Thus, 2fis 1×2, 1×3, 1×4, 2×3, 2×4, 3×4 are aliased with one other two-factor interaction, denoted by 1×2≈3×4, 1×3≈2×4, 2×3≈1×4. In this example, the confounding relationship between component and factor effects is one-to-one. However, this is a special case. In most three-level designs, the confounding relationships between the two are not necessarily one-to-one. For example, consider another $3^{5-2}$ design $D=\{1,\;2,\;3,\;123,12^{2}\}$ with the defining relation $I=1234^{2}=12^{2}5^{2}=13^{2}45=23^{2}45^{2}$. We have $12=34^{2}=15=25^{2}$, $12^{2}=5$, $13=24^{2}$, and $13^{2}=45$. Thus, 2fis 1×2≈3×4≈1×5≈2×5≈5, and 1×3≈2×4≈4×5. Note that in the aliasing relationship denoted by ≈, transitivity does not hold. Using the aforementioned aliasing relationships as an example, 1×2 is aliased with three 2fis: 3×4, 1×5, 2×5, and one main effect 5. However, it cannot be inferred that the 2fi 3×4 is aliased with 1×5, 2×5, or the main effect 5.

Next, we introduce a new classification pattern to describe the aliasing relationships among various factor effects in three-level designs. In the orthogonal component system, a *j*fi consists of $2^{j-1}$ mutually orthogonal *j*fics. Thus, an *i*fi is aliased with at most one component of a *j*fi, while the remaining *i*fics are not aliased with this *j*fi. Denote Kj=nj. An *i*fi is aliased with at most $K_{j}$ *j*fis. For 0≤i,j≤n and $0\leq k\leq K_{j}$, let Aj(k)i# be the number of *i*fis aliased with *k* *j*fis. The set {Aj(k)i#,i,j=0,1,2,…,n;k=0,1,2,…,Kj} is used to measure the aliasing degrees between various factor effects of any three-level designs. The larger *k* is, the more severe the aliasing in the set. Given the fixed value (i,j), Aj(k)i#(k=0,1,…,Kj) are arranged according to the aliasing severity, and denote Aj i#=(Aj(0)i#,Aj(1)i#,…,Aj(Kj)i#) for 0≤i,j≤n.

Based on the effect hierarchy principle, we introduce a factorial effect hierarchy principle (FEHP): (i) A lower-order factor effect is likely more important than a higher-order one, and (ii) factor effects of the same order are equally important. For any (i,j) and (s,t), the sorting rules are based on the FEHP: (i) If max{i,j}<max{s,t}, then Aj i# is ranked before At s#. (ii) If max{i,j}=max{s,t} and i<s, then Aj i# is ranked before At s#. (iii) If max{i,j}=max{s,t} and i=s, j<t, then Aj i# is ranked before At s#. Note that the 0th-order effect is the grand mean. For i≥2, we have A0 i#=(ni−Ai,Ai) and Ai 0#=(0Ai,1), where $A_{i}$ is the number of *i*fics aliased with the grand mean. We use $0^{s}$ to denote *s* successive zero components; the tail part is cut hereafter if it has a tail with successive zero components. Since A0 i# is determined from the preceding Ai 0#, A0 i# is ignored in the ranking process. According to the above ranking rules, we obtain a new sequence as follows:

(1) A   #=(A2 1#,A1 2#,A2 2#,A3 0#,A3 1#,A3 2#,A1 3#,A2 3#,A3 3#,…),

called the aliased factor-number pattern (AFNP) of a three-level design. For the abovementioned $3^{5-2}$ design, we have (A2 1#;A1 2#;A2 2#)= (2,3;7,3;0,0,6,4). Based on the definition of factor aliasing, the relationship ∑k=12kA1(k)2#=∑k=1K2kA2(k)1# holds. Thus, in analyzing the aliasing between main effects and 2fis, only A2 1# needs to be considered.

One of the main objectives of experimental design is to estimate as many factor effects as possible, particularly lower-order factor effects, such as main effects and 2fis. Therefore, a good design should minimize aliasing among lower-order factor effects and sequentially maximize the elements in ^#^*A*. Based on the AFNP, we propose a new optimal criterion as follows.

 **Definition 1.** *Let Al  # be the l-th component of ^#^A and ^#^$A\left(D_{i}\right)\left(i=1,2\right)$ be the AFNPs of designs $D_{i}\left(i=1,2\right)$. Suppose that ^#^$A_{l}$ is the first element such that ^#^$A\left(D_{1}\right)$ and ^#^$A\left(D_{2}\right)$ are different. If Al  #(D1)>Al  #(D2), then $D_{1}$ has less general lower-order aliasing based on factor effect (GLOAF) than $D_{2}$. A design D is said to have general minimum lower-order aliasing based on factor effects (GMLOAF, or GMAF for short) if no other design has less GLOAF than D, and such a design is a GMAF design.*

 **Example 1.** *Consider three $3^{8-4}$ designs $D_{i}\left(i=1,2,3\right)$. Table 1 shows the elements Aj i#(i,j=1,2) of their AFNPs. Thus, the optimal ordering of the designs under the GMAF criterion is $D_{1},D_{2},D_{3}$, whereas the ordering changes to $D_{3},D_{2},D_{1}$ under the GMC criterion. This indicates that the ranking results of designs vary significantly under different optimal criteria. The designs selected based on the GMAF criterion minimize aliasing among lower-order factor effects. Unlike the GMC criterion, which evaluates confounding at the components of a factor, GMAF considers the factor as a whole, thereby reducing computational complexity and improving the efficiency of identifying optimal factor combinations.*

Under the assumption that all three-order or higher-order effects are negligible, we only analyze two elements A2 1#(D) and A2 2#(D) of the AFNP. Let $F_{q}$ be a set of all possible pairwise combinations of elements in $H_{q}$, with its explicit structure:

$$F_{q}=\left\{a\times b|a,b\in H_{q},a\neq b\right\}.$$

For instance, $F_{2}=\left\{1\times 2,\;1\times \left(12\right),\;1\times \left(12^{2}\right),\;2\times \left(12\right),\;2\times \left(12^{2}\right),\;\left(12\right)\times \left(12^{2}\right)\right\}.$ Note that the number of elements in $H_{q}$ is given by $(3^{m}-1)/2$, and the number of elements in $F_{q}$ is (3m−1)/22. Let $\tilde{D}$ denote the factor structure of *D*. In a three-level design *D*, the number of 2fis aliased with $\eta \in \tilde{D}\cup F_{q}$ is defined as:

$$E_{2}\left(D,\eta \right)=\#\left\{\left(d_{1},d_{2}\right):d_{1}\times d_{2}\approx \eta ,\;d_{1},d_{2}\in D\right\},$$

where $d_{1}\times d_{2}$ represents the corresponding two-factor interaction. Thus, the following expressions of A2 1# and A2 2# are derived by

(2) A2(k)1#(D)=#{η:η∈D˜,E2(D,η)=k},A2(k)2#(D)=#{η:η∈Fq,E2(D,η)=k+1}

for $0\leq k\leq K_{2}$.

 **Example 2.** *Consider a $3^{5-2}$ design $D_{4}=\left\{1,2,3,123,12\right\}$, with its factor structure ${\tilde{D}}_{4}=\left\{1,2,3,1\times 2\times 3,1\times 2\right\}$. The confounding relationships between main effects and 2fics are given as follows:*

$$\begin{array}{cc} & 1=25^{2},\;2=15^{2},\;3=45^{2},\;4=35,\;5=12=34^{2},\\ & 13=24^{2},\;23=14^{2},\;25=12^{2}=15,\;35^{2}=45=34. \end{array}$$

We first calculate the values of A2(k)1# for $\eta \in \tilde{D}$ by the definition of $E_{2}\left(D,\;\eta \right)$. For $\eta \in \tilde{D}$, we have:

$$E_{2}\left(D,\;\eta \right)=\left\{\begin{array}{cc} 1, & \eta \in \{1,\;2,\;3,\;1\times 2\times 3\},\\ 2, & \eta =1\times 2. \end{array}\right.$$

Thus, A2(1)1#(D4)=#{η:η∈D˜4,E2(D4,η)=1}=4,A2(2)1#(D4)=#{η:η∈D˜4,E2(D4,η)=2}=1. For all other *k*, A2(k)1#(D4)=0. Then, A2 1#(D4)=(0,4,1). For $\eta \in F_{3}$, we have:

$$E_{2}\left(D,\;\eta \right)=\left\{\begin{array}{cc} 2, & \eta \in \{1\times 3,\;1\times (123),\;2\times 3,\;2\times (123)\},\\ 3, & \eta \in \{1\times (12),\;2\times (12),\;3\times (12),\;(123)\times (12)\},\\ 4, & \eta \in \{1\times 2,\;3\times (123)\}. \end{array}\right.$$

Hence, A2(1)2#(D4)=#{η:η∈F3,E2(D4,η)=2}=4,A2(2)2#(D4)=#{η:η∈F3,E2(D4,η)=3}=4, and A2(3)2#(D4)=2. For all other *k*, A2(k)2#(D4)=0. Therefore, A2 2#(D4)=(0,4,4,2).

## 3. Relationship with the Existing Criteria

### 3.1. Relationship with GMC Criterion

We review the GMC criterion proposed by Li et al. [10] for selecting optimal three-level designs. For 1≤i,j≤n, let Cj(k)j# be the number of *i*th-order effects confounded with *k* *j*th-order effects. Denote Cj i#=(Cj(0)i#,Cj(1)i#,…,Cj(kj)i#) for 0≤i,j≤n and Kj=nj. We call the simplified sequence

C   #=(C2 1#,C2 2#,C3 1#,C3 2#,C2 3#,C3 3#,…),

the ACNP of the design. A design that sequentially maximizes ^#^C is called a GMC design. Following Li et al. [10], for a $3^{n-m}$ design *D*, $B_{2}\left(D,\gamma \right)$ is the number of 2fics in *D*, confounded with $\gamma (\in H_{q})$, and defined as follows:

$$B_{2}\left(D,\gamma \right)=\#\left\{\left(d_{1},d_{2}\right):d_{1}d_{2}=\gamma \;\mathrm{or}\;d_{1}d_{2}^{2}=\gamma ,\;d_{1},d_{2}\in D\right\}.$$

Further, C2 1# and C2 2# are expressed as:

C2(k)1#(D)=#{γ:γ∈D,B2(D,γ)=k},C2(k)2#(D)=(k+1)#{γ:γ∈Hq,B2(D,γ)=k+1}.

Since every main effect only corresponds to a component, it follows that:

$$\#\left\{\gamma :\gamma \in D,\;B_{2}\left(D,\gamma \right)=k\right\}=\#\left\{\eta :\eta \in \tilde{D},\;E_{2}\left(D,\eta \right)=k\right\}.$$

That is to say, C2(k)1#(D)=A2(k)1#(D) always holds. More generally, Xj(k)1#(D)=Aj(k)1#(D) for any 0≤j≤n.

Based on the GMAF criterion, we analyze the relationship between the AFNP and the ACNP. If an ifi is aliased with *k* jfis, let $k_{t}$ represent the degree of confounding between the *t*-th component of the ifi and jfics, where $t=1,2,\;\ldots ,2^{i-1}$. It holds that $\sum_{t=1}^{2^{i-1}}k_{t}=k$. In the case of 2fis, each 2fi generates two components. Thus, for a $3^{n-m}$ design, the value of A2(k)1#(D) represents the number of $\eta \in F_{q}$ that are aliased with k+1 2fis, and is defined as

A2(k)1#(D)=#{η:η∈Fq,E2(D,η)=k1+k2+1}

with $k_{1}+k_{2}=k$. The terms $k_{1}$ and $k_{2}$ indicate the degrees to which the two 2fics of a given 2fi are confounded with other 2fics, and their sum reflects the overall degree of aliasing of the 2fi. Consequently, for each component, we have:

C2(k1)2#(D)=(k1+1)#{γ:γ∈Hq,B2(D,γ)=k1+1},C2(k2)2#(D)=(k2+1)#{γ:γ∈Hq,B2(D,γ)=k2+1}.

In the case that ifi and jfi are aliased, if the $2^{i-1}$ifics within ifi contain only one ific that is confounded with jfic, the component confounding and factor aliasing can be considered to have a one-to-one relationship. In this specific scenario, the AFNP and the ACNP are equivalent, that is, Cj(k)i#=Aj(k)i#(i,j=1,…,n). Next, we will analyze the relationship between the lower-order AFNP and ACNP in general cases.

 **Theorem 1.** *For a $3^{n-m}$ regular design with resolution R≥ III, the following results hold.*

 *(a)* *For k=0, we have C2(0)2#−2A2(0)2#=#{η:η∈Fq,E2(D,η)=k2+1,k2≥1}.*
 *(b)* *If C2(k)2#>A2(k)2# for k≥1, then*
C2(k)2#−A2(k)2#=#{η:η∈Fq,E2(D,η)=k+k2+1,1≤k2≠k}+2#{η:η∈Fq,E2(D,η)=2k+1,k1=k2=k}.
 *(c)* *If C2(k)2#<A2(k)2# for k≥2, then *
A2(k)2#−C2(k)2#=#{η:η∈Fq,E2(D,η)=k1+k2+1,k1+k2=k,k1,k2>0}

 **Proof.** (a) According to the definition of factor aliasing, if a 2fi is not aliased with other 2fis, its components are also not confounded with any other 2fics. However, a 2fi may have one component confounded with other 2fics while the other remains unconfounded. This scenario leads to the inequality C2(0)2#≥A2(0)2#.

To quantify this phenomenon, we use C2(0)2#−2A2(0)2# to represent the number of 2fics within 2fis that are aliased with other 2fis but, at the same time, are not themselves confounded with any other 2fics. For such 2fis, there exists exactly one 2fic that is not confounded with any other 2fics. Without loss of generality, assume that the first 2fic of such a 2fi is unconfounded with other 2fics, i.e., let $k_{1}=0$. Under this assumption, the number of 2fics satisfying the above description can be expressed as:

C2(0)2#−2A2(0)2#=#{η:η∈Fq,E2(D,η)=k2+1,k2≥1}.

(b) In the case of 2fis aliased with *k* other 2fis, if one of the two 2fic components of the 2fi is confounded with *k* other 2fics while the other 2fic is not confounded with any other 2fics, then C2(k)2#=A2(k)2#.

If a 2fi is aliased with other 2fis to a degree strictly greater than *k*, two particular 2fics involving this 2fi can be identified. One of these 2fics is confounded with exactly *k* other 2fics, while the other, although also confounded with this 2fic, has a confounding degree with other 2fics that is not equal to *k*. The number of such 2fics is expressed as:

$$\#\{\eta :\eta \in F_{q},\;E_{2}\left(D,\;\eta \right)=k+k_{2}+1,\;1\leq k_{2}\neq k\}.$$

On the other hand, if both 2fics of the 2fi are confounded with *k* other 2fics, the difference value of C2(k)2#−A2(k)2# reflects the number of 2fics within 2fis whose aliasing degree is 2k. These 2fics are also confounded with *k* other 2fics. The number of 2fics satisfying this condition can be expressed as:

$$2\#\{\eta :\eta \in F_{q},\;E_{2}\left(D,\;\eta \right)=2k+1,\;k_{1}=k_{2}=k\}.$$

Therefore, when C2(k)2#>A2(k)2#, the corresponding difference equals the total number of 2fics within 2fis aliased with more than *k* other 2fis, where the degree of confounding with 2fics is *k*. This total number can be expressed as:

C2(k)2#−A2(k)2#=  #{η:η∈Fq,E2(D,η)=k+k2+1,1≤k2≠k}+2#{η:η∈Fq,E2(D,η)=2k+1,k1=k2=k}.

In this scenario, at least one 2fic must be confounded with *k* other 2fics. Thus, k≥1.

(c) When C2(k)2#<A2(k)2#, the aliasing degree *k* can be achieved in multiple ways. If a 2fi has only one 2fic confounded with *k* other 2fics, while the remaining 2fic is not confounded with any other 2fi, then the aliasing degree *k* is entirely determined by this single component. In this scenario, the factor aliasing and component confounding correspond individually.

When at least two components determine the aliasing degree *k*, the difference value of A2(k)2#−C2(k)2# represents a situation that, among the 2fis aliased with *k* other 2fis, the degree *k* is jointly determined by exactly two 2fics. The number of 2fis fitting this description can be expressed as:

A2(k)2#−C2(k)2#=#{η:η∈Fq,E2(D,η)=k1+k2+1,k1+k2=k,k1,k2>0}.

Note that the aliasing degree *k* can only be determined by a single component when k=1. Therefore, the situation Cj(1)i#≥Aj(1)i# can only occur when k≥2.    □

We provide an example to illustrate Theorem 1.

 **Example 3.** *Consider a $3^{6-2}$ design $D_{5}=\left\{1,2,3,4,1234,12^{2}3\right\}$. Based on the AFNP and ACNP, its lower-order aliasing indices are (A2 1#;A2 2#)=(6;4,10,1) and C2 2#=(18,12). The specific confounding patterns of the 2fics are as follows:*

$$\begin{array}{c} 13=26,23^{2}=16^{2},24^{2}=56^{2},26^{2}=45^{2},46=25,36^{2}=12^{2},56^{2}=24. \end{array}$$

According to Theorem 1, C2(0)2#−2A2(0)2#=10. Thus, there are 10 2fis with two components, one of which is confounded with the other 2fics, and the other is not. These 2fis are 1×2, 1×3, 1×6, 2×3, 2×4, 2×5, 3×6, 4×5, 4×6, 5×6. From C2(1)2#−A2(1)2#=2, it can be concluded that among the 2fis aliased with at least one other 2fi, two of their components, 26 and $26^{2}$, are each confounded with one other 2fic. Finally, from A2(2)2#−C2(2)2#=1, it follows that there exists a 2fi aliased with two other 2fis, and its aliasing degree is determined by two components. The 2fi satisfying this case is 2×6.

The definitions of the GMAF and GMC criteria clearly show that the GMAF criterion imposes stricter requirements for optimal designs than the GMC criterion. The GMAF criterion focuses on the overall aliasing of factor effects rather than the factor effect components.

### 3.2. Relationship with Entropy

To adapt to the entropy formulation, in a $3^{n-m}$ design, denote the factors as $d_{1},d_{2},\;\ldots ,d_{n}$, where $d_{1},\;\ldots ,d_{n-m}$ represent independent factors (i.e., independent columns), and the remaining $d_{n-m+1},\;\ldots ,d_{n}$ are generator factors (i.e., additional columns). We employ entropy to measure factor uncertainty. Let F denote all possible combinations of factor values, and p(d) represent the estimated probability of occurrence for a particular combination d∈F [17,18]. The entropy of the design matrix is defined as

$$H\left(d\right)=-\sum_{d\in F}p\left(d\right)\log p\left(d\right).$$

In fractional factorial designs, to assess the degree of aliasing among factors, we focus on the conditional entropy of generators with respect to other factors, such as $H(d_{i}|d_{1},\;\ldots ,d_{i-1},d_{i+1},\;\ldots ,d_{m})$. Lower conditional entropy indicates stronger dependence of the factor on other generators, implying more severe aliasing. Specifically, if a factor is a function of other generators, its conditional entropy equals zero, indicating aliasing. Conversely, higher conditional entropy suggests that the factor retains uncertainty given other factors, indicating less aliasing.

 **Example 4.** *Consider two $3^{5-1}$ designs: $D=\{d_{1},d_{2},d_{3},d_{4},d_{5}=d_{1}d_{2}\}$ and $D^{\prime }=\{d_{1},d_{2},d_{3},d_{4},$$d_{5}=d_{1}d_{2}d_{3}\}$, where $d_{5}$ is the only generated factor. In design D, the conditional entropy $H(d_{5}|d_{1},d_{2})=0$, indicating that $d_{5}$ is fully determined by $d_{1}$ and $d_{2}$, and thus entirely confounded with them. In contrast, for $D^{\prime }$, although $H(d_{5}|d_{1},d_{2},d_{3})=0$, it holds that $H(d_{5}|d_{1},d_{2})>0$, meaning that $d_{5}$ retains some uncertainty given only $d_{1}$ and $d_{2}$. This implies that design $D^{\prime }$ has a lower degree of aliasing compared to D.*

Therefore, for a fixed number of factors *n* and generators *m* with the same number of levels, we can compare the conditional entropies associated with generators across designs to evaluate the confounding severity. Specifically, under the same conditions, a larger conditional entropy indicates lower confounding among factors and higher resolution. Consequently, the designs selected based on the conditional entropy criterion yield the same results as those obtained under the MR criterion. However, the conditional entropy criterion exhibits an inherent limitation; it cannot differentiate among multiple designs with same resolution. Based on Definition 1, we can directly obtain the following theorem.

 **Theorem 2.** *A GMAF $3^{n-m}$ design must have maximum resolution among all $3^{n-m}$ designs, and exhibit the minimum factor aliasing among all $3^{n-m}$ designs with the same conditional entropy.*

According to this theorem, the GMAF criterion overcomes the inherent limitations of the conditional entropy criterion. GMAF designs not only satisfy the maximum resolution criterion, but also minimize factor aliasing among all designs with the same conditional entropy. This provides a more precise discrimination for comparing designs with the same conditional entropy.

### 3.3. Relationship with MA Criterion

For a $3^{n-m}$ design, the set of all possible products of the *m* words generates a defining contrast subgroup *G*. Let $A_{i}$ (i=1,…,n) denote the number of words of length *i* in *G*. The vector $W=(A_{1},A_{2},\;\ldots ,A_{n})$ is called the WLP. A design sequentially minimizing the vector *W* is considered an MA design. To study the relationship between the GMAF and MA criteria, we investigate the relationship between the WLP and the AFNP as the cores of MA and GMAF criteria, respectively.

 **Theorem 3.** *For a $3^{n-m}$ regular design with a resolution R≥III, we have:*

Ai=1i∑k=1Ki−1kAi−1(k)1#−2n+2−iiAi−2−i−1iAi−1=1i∑k=12i−2ki#A1(k)−1 −2n+2−iiAi−2−i−1iAi−1,i≥3.

 **Proof.**  If i≥3, it is known from Zhang and Mukerjee [9] that:

∑k=1Ki−1kCi−1(k)1#=2(n−i+2)Ai−2+(i−1)Ai−1+iAi.

Since Ci−1(k)1#=Ai−1(k)1#, then by keeping $iA_{i}$ on the right-hand side, we have:

iAi=∑k=1Ki−1kAi−1(k)1#−2(n−i+2)Ai−2−(i−1)Ai−1.

Dividing both sides by *i* yields:

Ai=1i∑k=1Ki−1kAi−1(k)1#−2n+2−iiAi−2−i−1iAi−1.

By rearranging this equation, the value of $A_{i}$ can be expressed as a weighted sum of ∑k=1Ki−1kAi−1(k)1#, $A_{i-2}$, and $A_{i-1}$. Moreover, since the resolution R≥III, the main effects are not aliased with other main effects, and the following equivalence holds: ∑k=1Ki−1kAi−1(k)1#=∑k=12i−2ki#A1(k)−1 . In particular, it follows that $A_{1}=A_{2}=0$ when R≥III.    □

Through calculations, the relationship between the three-level WLP and AFNP is derived as follows:

A3=13∑k=1K2kA2(k)1#=13∑k=12kA1(k)2#,A4=14∑k=1K3kA3(k)1#−34A3=14∑k=122kA1(k)3#−34A3=14∑k=1K3kA3(k)1#−14∑k=1K2kA2(k)1#,A5=15∑k=1K4kA4(k)1#−2n−35A3−45A4=15∑k=123kA1(k)4#−2n−35A3−45A4=15∑k=1K4kA4(k)1#−15∑k=1K3kA3(k)1#+9−2n15∑k=1K2kA2(k)1#.

For example, a $3^{5-2}$ design $D_{6}=\left\{1,2,3,12,12^{2}\right\}$ have A1 2#=(4,0,6). Hence, A3=13∑k=12kA1(k)2#=4.

Note that a design includes *k*ifics from the same ifi in its independent defining relation, which necessarily results in A1(k)i#≠0 when k≥2. Based on the WLP, the designs with different WLPs may have different AFNPs. Minimizing the WLP sequentially is equivalent to maximizing {A0 i#,i=3,…,n} or {Ai 0#,i=3,…,n} sequentially. However, the different AFNPs may have the same WLP.

 **Example 5.** *Consider the two $3^{13-8}$ designs:*

$$\begin{array}{cc} D_{7} & =\{1,\;2,\;3,\;4,\;5,\;12345,\;12^{2}3^{2}4,\;12^{2}4^{2}5,\;23^{2}4^{2}5,\;123^{2}5^{2},\;12^{2}3,\;124,\;125\},\\ D_{8} & =\{1,\;2,\;3,\;4,\;5,\;12345,\;12^{2}3^{2}4,\;12^{2}4^{2}5,\;23^{2}4^{2}5,\;123^{2}5^{2},\;12^{2}3,\;124,\;13^{2}45\}. \end{array}$$

The AFNPs of designs $D_{7}$ and $D_{8}$ are different. In particular, they first differ at A2(1)1#(D7)=5 and A2(1)1#(D8)=3. However, they share the same $W=\left(A_{3},\;A_{4},\;A_{5},\;A_{6}\right)=\left(0,\;24,\;108,\;207\right)$.

Consequently, the AFNP is a more refined pattern than the WLP since the WLP is only related to the elements {A0(1)i#,i=1,2,…,n} of the AFNP. Consider two design:

$$\begin{array}{cc} \; & D:I=123456^{2}=12^{2}3^{2}47^{2}=12^{2}4^{2}58^{2}=12^{2}3^{2}9^{2}=13^{2}5^{2}t_{0}^{2}=134^{2}t_{1}^{2}=23^{2}4^{2}t_{2}^{2},\\ \; & D^{\prime }:I=123456^{2}=12^{2}3^{2}47^{2}=12^{2}4^{2}58^{2}=23^{2}4^{2}59^{2}=123^{2}5^{2}t_{0}^{2}=12^{2}3t_{1}^{2}=124t_{2}^{2}, \end{array}$$

where $t_{0},t_{1},t_{2}$ represent factors 10, 11, and 12, respectively. Under the MA criterion, the design *D* is better than $D^{\prime }$. However, according to the GMAF criterion, the design $D^{\prime }$ is optimal. Since the MA criterion uses only part of the information in the AFNP, the optimal design under the MA criterion is not as good as that obtained by the GMAF criterion.

### 3.4. Relationship with CE Criterion

For a three-lever design, a main effect or 2fi is called clear if it is not aliased with other main effects or 2fis. We study the relationship between the CE and GMAF criteria by calculating the number of clear effects using the AFNP. Let $C_{1}$, $C_{2}$, and CC be the number of clear main effects, clear 2fis, and clear 2fics, respectively.

 **Theorem 4.** *For any three-level design with resolution III or higher, we have:*

C1=A2(0)1#,C2=A2(0)2#.

 **Proof.**  For three-level designs with resolution III or higher, any main effect is not aliased with other main effects. The A2(0)1# is just the number of main effects that are aliased with neither any main effect nor any 2fi, that is, C1=A2(0)1#.

Consider a 2fi A×B, with its two 2fics AB and $AB^{2}$ not confounded with any other 2fics. Suppose AB or $AB^{2}$ is confounded with a main effect. In a defining word of length 3, if AB or $AB^{2}$ is confounded with a main effect, the defining word must include both factors *A* and *B*. This would inevitably lead to AB or $AB^{2}$ being confounded with other 2fics, contradicting the initial assumption. Therefore, if the two components of a 2fi are not confounded with any other 2fics, then the 2fi is clear. Based on the definition of A2(0)2#, it represents the number of 2fis that are not aliased with any other 2fis. Thus, C2=A2(0)2#.    □

Therefore, in the factor aliasing of three-level designs, the CE criterion selects designs that sequentially maximize $C_{1}$ and $C_{2}$. According to the definitions of clear 2fis and clear 2fics, the difference value between CC and $C_{2}$ represents the number of 2fics within 2fis aliased with other 2fis but not with any other 2fics. Specifically, this relationship can be expressed as:

CC−2C2=C2(0)2#−C2(1)1#−2C2(0)2#.

The inequality $CC-2C_{2}\geq 0$ implies that, in general, the number of clear 2fics is greater than or equal to the number of clear 2fis. This follows from the fact that a 2fi is considered clear only if both of its 2fics are clear. Therefore, the criterion for a clear 2fi is inherently more stringent than that for individual components. From a design perspective, the stricter requirement for clarity in factor effects better aligns with the practical need to accurately identify significant factor effects in experimental settings.

The following results are obtained based on Theorems 1 and 3 in Ai and Zhang [19].

 **Theorem** **5.** *(i) If $n\leq (3^{q-1}-1)/2+1$, a $3^{n-m}$ design with sequentially maximizing A2(0)1# and A2(0)2# is an optimal design under CE criterion.*

*(ii) If there exists an optimal $3^{n-m}$ design with resolution IV under the CE criterion, then the GMAF design must be the best one among all optimal designs under the CE criterion, where the meaning of ’best’ is under the comparison in Definition 1 of the GMAF criterion.*

However, the CE criterion cannot distinguish designs with the same number of clear main effects and 2fis, while the GMAF criterion can. The following example illustrates this point.

 **Example 6.** *Consider the type of $3^{7-3}$ designs. According to Xu [20], there are 19 non-isomorphic designs. Under the CE criterion, the optimal designs are $D=\{1,2,3,4,134^{2},12^{2}3,13^{2}4\}$ and $D^{\prime }=\left\{1,2,3,4,1234,12^{2}3,12^{2}4\right\}$. Both designs have 7 clear main effects and no clear 2fis. However, A2 2#(D)=(0,12,9), and A2 2#(D′)=(0,6,15). Under the GMAF criterion, the design D is better than the design $D^{\prime }$. It can be concluded that the optimal design under the GMAF criterion represents the best-performing design among all optimal designs under the CE criterion.*

## 4. Aliasing Algorithm of Lower-Order Factors

Factor aliasing patterns are crucial in selecting optimal designs, especially in aliasing lower-order factors. This section mainly introduces some algorithms to calculate the lower-order factor aliasing. The algorithm is based on defined contrast subgroups. It includes the following three steps: (i) generate a design matrix from a saturated design, (ii) construct the defining contrast subgroup matrix, and (iii) calculate the low-order factor aliasing values of the design. The specific implementation code is available in the Supplementary Materials.

### 4.1. Generate Design Matrix

Let M(D) be the design matrix of a $3^{n-m}$ design *D*. The matrix M(D) is generated from the corresponding saturated design $H_{q}$, which each component is expressed as ${\left(b_{1},b_{2},\;\ldots ,b_{q}\right)}^{\prime }$, where $b_{i}\in GF\left(3\right)$ and $b_{1},b_{2},\;\ldots ,b_{q}$ are not all zero. Proportional components are considered equivalent. A vector $b={\left(b_{1},\;\ldots ,b_{n}\right)}^{\prime }$ can be denoted as $1^{b_{1}}2^{b_{2}}\ldots n^{b_{n}}$, with the convention that $i^{b_{i}}$ is omitted for $b_{i}=0$. For example, in $H_{3}$, 12 corresponds to the vector (1,1,0), while in $H_{4}$, it is represented as (1,1,0,0). In the following, we present an algorithm for generating the design matrix.

 **Example 7.** *Consider a $3^{5-2}$ design with index set D={1,2,5,8,4}. The design matrix M(D) is constructed by extracting columns 1, 2, 5, 8, and 4 from the saturated design matrix $H_{3}$. Using Algorithm 1, we obtain:*

$$\begin{array}{c} H_{3}=\left[\begin{array}{ccccccccccccc} 1 & 0 & 1 & 1 & 0 & 1 & 0 & 1 & 1 & 1 & 0 & 1 & 1\\ 0 & 1 & 1 & 2 & 0 & 0 & 1 & 1 & 2 & 0 & 1 & 1 & 2\\ 0 & 0 & 0 & 0 & 1 & 1 & 1 & 1 & 1 & 2 & 2 & 2 & 2 \end{array}\right],\;M\left(D\right)=\left[\begin{array}{ccccc} 1 & 0 & 0 & 1 & 1\\ 0 & 1 & 0 & 1 & 2\\ 0 & 0 & 1 & 1 & 0 \end{array}\right]. \end{array}$$

The saturated matrix $H_{3}$ has 13 columns, each of which can be represented as a component. The first and second columns of $H_{3}$ are independent components. Using addition modulo 3 over GF(3), the interaction of the first and second columns produces two components, 12 and $12^{2}$, represented by the third and fourth columns of $H_{3}$, respectively. According to the design column index set D={1,2,5,8,4}, the 1st, 2nd, 5th, 8th, and 4th columns are selected from $H_{3}$ to form the design matrix M(D).


**Algorithm 1:** Generate design matrix M(D)

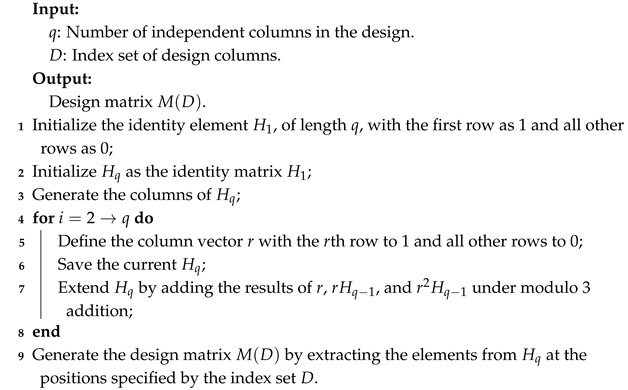




### 4.2. Construct Defining Contrast Matrix

Through Algorithm 1, we can obtain the additional column matrix *F* from the design matrix M(D). Next, we calculate the defining contrast matrix M(G) under modulo 3 addition in Algorithm 2.


**Algorithm 2:** Construct defining contrast matrix M(G)

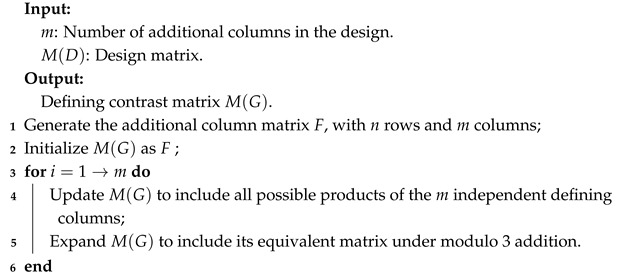




In Example 7, the two columns of *F* represent $1234^{2}$ and $12^{2}5^{5}$. Subsequently, we construct a defining contrast matrix M(G) under modulo 3 addition, which contains eight elements, including the modulo 3 equivalent components for each element.

$$\begin{array}{c} F=\left[\begin{array}{cc} 1 & 1\\ 1 & 2\\ 1 & 0\\ 2 & 0\\ 0 & 2 \end{array}\right],\; \end{array}M\left(G\right)=\left[\begin{array}{cccccccc} 1 & 1 & 2 & 0 & 0 & 1 & 2 & 2\\ 1 & 2 & 0 & 2 & 1 & 0 & 2 & 1\\ 1 & 0 & 1 & 1 & 2 & 2 & 2 & 0\\ 2 & 0 & 2 & 2 & 1 & 1 & 1 & 0\\ 0 & 2 & 2 & 1 & 2 & 1 & 0 & 1 \end{array}\right].$$

### 4.3. Calculate the Lower-Order Factor Aliasing

Based on the FEHP, lower-order factor effects are more important than high-order ones. An algorithm is used to calculate A2 1# and A2 2# for any $3^{n-m}$ design *D*. These results are instrumental in identifying GMAF designs. The detailed steps of the algorithm are outlined below.

Algorithm 3 provides a method for calculating lower-order factor aliasing based on the defining contrast matrix M(G). For Example 7, we first generate the corresponding 2fic matrix B(5) as follows:

$$B\left(5\right)=\left[\begin{array}{cccccccccccccccccccc} 1 & 1 & 1 & 1 & 1 & 1 & 1 & 1 & 0 & 0 & 0 & 0 & 0 & 0 & 0 & 0 & 0 & 0 & 0 & 0\\ 1 & 2 & 0 & 0 & 0 & 0 & 0 & 0 & 1 & 1 & 1 & 1 & 1 & 1 & 0 & 0 & 0 & 0 & 0 & 0\\ 0 & 0 & 1 & 2 & 0 & 0 & 0 & 0 & 1 & 2 & 0 & 0 & 0 & 0 & 1 & 1 & 1 & 1 & 0 & 0\\ 0 & 0 & 0 & 0 & 1 & 2 & 0 & 0 & 0 & 0 & 1 & 2 & 2 & 0 & 1 & 2 & 0 & 0 & 1 & 1\\ 0 & 0 & 0 & 0 & 0 & 0 & 1 & 2 & 0 & 0 & 0 & 0 & 0 & 2 & 0 & 0 & 2 & 2 & 1 & 2 \end{array}\right].$$

The elements in the $B_{split}\left(5\right)$ are written as follows:

$$B_{1}=\left\{{\left(1,1,0,0,0\right)}^{\prime },{\left(1,2,0,0,0\right)}^{\prime }\right\},\;\ldots ,B_{10}=\left\{{\left(0,0,0,1,1\right)}^{\prime },{\left(0,0,0,1,2\right)}^{\prime }\right\}.$$

**Algorithm 3:** Calculate A2 1# and A2 2#

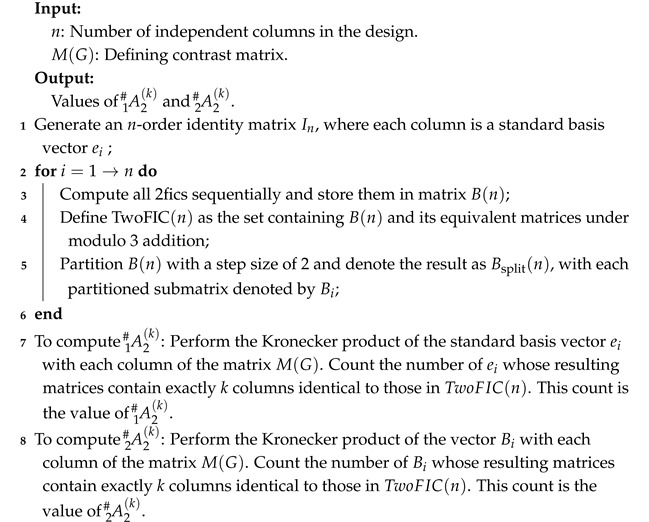




Subsequently, each $e_{i}\;\left(i=1,2,\;\ldots ,5\right)$ and $B_{i}\;\left(i=1,2,\;\ldots ,10\right)$ is combined with the columns of M(G) under addition modulo 3 over GF(3) to perform the Kronecker product. The result is A2 1#=(2,3), indicating that two main effects are not aliased with any 2fi, and the remaining three main effects are each aliased with one other 2fi. Among the 10 2fis, six are aliased with two other 2fis, and the remaining four are aliased with three other 2fis, represented as A2 2#=(0,0,6,4).

### 4.4. A Catalog of Three-Level Designs with Lower-Order AFNP

Based on the aforementioned aliasing algorithm, the lower-order AFNP of all the 27-, 81-run designs with factor numbers n=5,…,20, and 243-run designs with resolution IV or higher are listed in Table 2, Table 3 and Table 4. For a design $3^{n-m}$, denoted as n−m.i, we only list the additional columns (abbreviated as add. columns), where *i* indicates the ranking of the design under the GMAF criterion. Here, the n−m.1 design is always the GMAF design. The first column of the table represents the selected design, and the second column represents the positions in the Yates order. The table also includes W= ($A_{3}$, $A_{4}$, $A_{5}$, $A_{6}$), $C_{1}$, $C_{2}$, CC, and the two primary components A2 1# and A2 2# of the AFNP, along with the two primary components C2 1# and C2 2# of the ACNP. The designs are ranked under the GMC and MA criteria at the end.

Table 2 presents all 27-run designs. The results show that each GMAF design is also a GMC and MA design, with equal rankings under both GMAF and GMC criteria. This indicates that the GMAF designs with 27 runs retain the advantageous properties of both GMC designs and MA designs. Moreover, the rankings of designs 5-2.2 and 5-2.3 under the GMC and MA criteria are 2, 3, and 3, 2, respectively. Similarly, for designs 6-3.3 and 6-3.4, the rankings under the GMC and MA criteria are 3, 4, and 4, 3, respectively. The rankings of the remaining 23 designs are consistent under the GMAF, GMC, and MA criteria.

For 81-run designs, there can be up to 40 columns. Table 3 lists the WLP, the number of clear main effects and clear 2fis, the AFNP, and the ACNP of some 81-run designs. As Xu [20] noted, any 27-run design can be considered a degenerate 81-run design. The number of non-isomorphic designs for n=21,…,35 is equal to the number of non-isomorphic designs for n=5,…,19. Thus, only 81-run designs with m=5,…,20 are listed. It can be observed that the GMAF designs for the 81-run are not necessarily GMC or MA designs. The results are summarized as follows. In the 81-run designs, among the optimal designs selected under the GMAF criterion: (i) Only designs 7-3.1 and 8-4.1 are not optimal under the GMC criterion, while the remaining designs are optimal under both the GMAF and GMC criteria. (ii) Only designs 12-8.1, 13-9.1, 14-10.1, 15-11.1, and 16-12.1 are not optimal under the MA criterion, while the remaining designs are optimal under both the GMAF and MA criteria.

For 243-run designs, there can be a maximum of 121 columns. Table 4 lists some 243-run designs. Following Xu [20], each 243-run design with a resolution of at least IV has a maximum of 20 columns. We only list 243-run designs with n=6,…,20. It can be observed that in the 243-run designs, when the number of independent columns is fewer than 7, designs 6-1.1, 7-2.1, 8-3.1, 9-4.1, 10-5.1, and 11-6.1 are optimal under the GMAF, GMC, and MA criteria. However, when the number of independent columns exceeds 7, designs 13-8.1, 14-9.1, 15-10.1, 16-11.1, 17-12.1, 18-13.1, 19-14.1, and 20-15.1 are optimal only under the GMAF criterion.

## 5. A Real Example

This section provides a seatbelt experiment to illustrate the effectiveness of the AFNP and the GMAF criterion [6]. In the study, we mainly investigate the aliasing properties of four factors on the pull strength of truck seat belts. The four factors are hydraulic pressure of crimping (1), die flat middle setting (2), length of crimp (3), and anchor lot (4), with each factor at three levels.

Since three-level regular designs are run size economy, we try to select an optimal $3^{4-1}$ design under the GMAF and other criteria. For $3^{4-1}$ designs, there are only two non-isomorphic $3^{4-1}$ designs $D_{1}=\left\{1,2,3,12\right\}$ and $D_{2}=\left\{1,2,3,123\right\}$, determined by the defining relations 4=12 and 4=123, respectively. The set ${\tilde{D}}_{i}\left(i=1,2\right)$ is the factor structure of $D_{i}\left(i=1,2\right)$. By calculation, we obtain the confounded relations between the main effects and 2fics of the design $D_{1}$ as follows:

$$\begin{array}{c} 1=24^{2},2=14^{2},4=12,14=12^{2}=24. \end{array}$$

For $\eta \in {\tilde{D}}_{1}\cup F_{3}$, we have:

$$E_{2}\left(D_{1},\;\eta \right)=\left\{\begin{array}{cc} 0, & \mathrm{if}\;\eta =3,\\ 1, & \mathrm{if}\;\eta \in \{1,\;2,\;1\times 2\}. \end{array}\right.E_{2}\left(D_{1},\eta \right)=\left\{\begin{array}{cc} 1, & \mathrm{if}\;\eta \in \{1\times 3,\;2\times 3,\;3\times (12)\},\\ 3, & \mathrm{if}\;\eta \in \{1\times 2,\;1\times (12),\;2\times (12)\}. \end{array}\right.$$

From (2), it yields that (A2 1#(D1);A2 2#(D1))=(1,3;3,0,3). Similarly, $E_{2}\left(D_{2},\;\eta \right)=0$ when $\eta \in {\tilde{D}}_{2}$. For $\eta \in \left\{1\times 2,\;1\times 3,\;1\times \left(12\right),\;2\times 3,\;2\times \left(123\right),\;3\times \left(123\right)\right\}\subset F_{3}$, we have $E_{2}\left(D_{2},\;\eta \right)=2$. Thus, (A2 1#(D2);A2 2#(D2))=(4;0,6). We observe that A2 1# is the first element, which makes ^#^*A* (*D*1) ≠ ^#^*A* (*D*2). Thus, the design $D_{2}$ is a GMAF design.

Under the GMC criterion, we need to calculate C2 1# and C2 2#. Based on the relationship of the AFNP and ACNP, we have C2 1#(Di)=A2 1#(Di),i=1,2. That is to say, C2 1#(D1)=(1,3), and C2 1#(D2)=(4,0). According to Theorem 1, we have

C2(0)2#(D1)=2A2(0)2#+#{η:η∈F3,E2(D1,η)=k2+1,k2≥1}=9,C2(1)2#(D1)=#{η:η∈F3,E2(D1,η)=1+k2+1,k2≠1}+2#{η:η∈F3,E2(D1,η)=2+1,k1=k2=1}=0,C2(2)2#(D1)=#{η:η∈F3,E2(D1,η)=2+k2+1,k2≠2}+2#{η:η∈F3,E2(D1,η)=4+1,k1=k2=2}=3.

For any other *k*, C2(k)2#(D1)=0. Hence, we have C2 2#(D1)=(9,0,3). Similarly, for the design $D_{2}$, we have C2 2#(D2)=(6,6). Then, the design $D_{2}$ is also a GMC design.

To compare the WLP and the number of clear factors between the two designs, it can be derived from Theorem 3 that:

A3(D1)=13∑k=1K2kA2(k)1#(D1)=1;A4=14∑k=1K3kA3(k)1#(D1)−14∑k=1K2kA2(k)1#(D1)=0,A3(D2)=13∑k=1K2kA2(k)1#(D2)=0;A4=14∑k=1K3kA3(k)1#(D2)−14∑k=1K2kA2(k)1#(D2)=1.

According to Theorem 4, we have C1(D1)=A2(0)1#(D1)=1, C2(D1)=A2(0)2#(D1)=3, C1(D1)=A2(0)1#(D2)=4, and C2(D2)=A2(0)2#(D2)=0.

Consider the two $3^{n-m}$ designs described above: design $D_{1}=\left\{d_{1},d_{2},d_{3},d_{4}=d_{1}d_{2}\right\}$, where factor $d_{4}$ is determined by $d_{1}$ and $d_{2}$, and design $D_{2}=\left\{d_{1},d_{2},d_{3},d_{4}=d_{1}d_{2}d_{3}\right\}$, where factor $d_{4}$ is determined by $d_{1}$, $d_{2}$, and $d_{3}$. In both designs, the individual entropies of the first three factors $d_{1},d_{2},d_{3}$ are approximately $H(d_{i})\approx 0.918$ bits (corresponding to the case where, among three levels, one level appears once and another appears twice), indicating a relatively balanced level distribution. We examine the conditional entropy of factor $d_{4}$ to reveal its aliasing relationships with other factors. In design $D_{1}$, since $d_{4}=d_{1}d_{2}$, its conditional entropy satisfies $H(d_{4}|d_{1},d_{2})=0$. This means that once $d_{1}$ and $d_{2}$ are given, the value of $d_{4}$ is completely determined with no remaining uncertainty, indicating that $d_{4}$ is confounded with $d_{1}$ and $d_{2}$. In design $D_{2}$, where $d_{4}=d_{1}d_{2}d_{3}$, we have $H(d_{4}|d_{1},d_{2},d_{3})=0$. However, the key difference is that $H(d_{4}|d_{1},d_{2})>0$. This indicates that when only $d_{1}$ and $d_{2}$ are known, $d_{4}$ retains some uncertainty. Therefore, $H\left(d_{4}|d_{1},d_{2}\right)\left(D_{2}\right)>H\left(d_{4}|d_{1},d_{2}\right)\left(D_{1}\right)$, demonstrating that design $D_{2}$ has less aliasing compared to design $D_{1}$, which aligns with the difference in design resolution.

The entropy analysis confirms the validity of the selected designs. Moreover, design $D_{2}$ is better than design $D_{1}$ under the MA, CE, GMC, and GMAF criteria. Thus, design $D_{2}$ should be selected for the experiment, with factors *A*–*D* assigned to its four columns. The WLP, along with $C_{1}$ and $C_{2}$, is the core of the MA and CE criteria, and all are functions of the AFNP, which offers more comprehensive information on factor aliasing compared to the WLP and $C_{1},C_{2}$. Additionally, the GMAF criterion retains the advantages of GMC, MA, and CE criteria while measuring aliasing from the factors’ perspective, thereby reducing computational complexity. This indicates that the GMAF criterion is practical and applicable for optimizing and selecting experimental designs.

## 6. Conclusions

This paper introduces the AFNP, which mainly uses three-level regular designs, and proposes the GMAF criterion. The AFNP’s main advantage embodies several aspects. First, the classification pattern contains all aliasing information between various factors of any three-level design. Based on the FEHP, assuming higher-order interactions can be ignored, $E_{2}\left(D,\eta \right)$ is defined to represent the number of 2fis aliased with η in a design *D*. For different values of η within the set *D* or $F_{q}$, $E_{2}\left(D,\eta \right)$ is used to represent both A2(k)1# and A2(k)2#, which facilitates the establishment of relationships between the GMAF criterion and other criteria. For instance, Theorem 1 demonstrates the quantitative relationship between C2(k)2# and C2(k)2#, establishing the connection between the GMAF and GMC criteria. Theorem 2 shows that the GMAF criterion overcomes the limitation of the conditional entropy criterion in evaluating designs with the same conditional entropy. Theorems 3 and 4 separately explore the relationships of the GMAF criterion with the MA and CE criteria. The MA criterion utilizes information from {Aj(k)i#|i,j=0,1,…,n,k=1,…,Kj}, whereas the CE criterion relies solely on {Aj(0)i#|i,j=0,1,…,n}. The GMAF criterion provides a more precise method for selecting designs. It overcomes the limitation of the MA criterion, which becomes inapplicable when the WLPs are identical. It outperforms the CE criterion by distinguishing optimal designs when the $C_{1}$ and $C_{2}$ parameters are equal. The results show that the core of other criteria is the specific functions of the AFNP. Then, an algorithm based on the definition of contrast subgroups is proposed to calculate lower-order factor aliasing, enabling the search for GMAF designs. The tables show catalogs of all 27-run designs, selected 81-run designs, and 243-run designs with resolutions higher than IV under the GMAF criterion. Finally, using the seatbelt experiment as an example, it is demonstrated that the GMAF criterion is practical and effective in its application.

This paper only focuses on the AFNP and GMAF criteria of three-level designs. The criterion can be extended to higher-level regular, blocked, and mixed-level designs. Additionally, how to construct a GMAF design needs to be further investigated, which is more complex than the topics discussed here and remains an open problem.

## Figures and Tables

**Table 1:** Some Aj′i#s of designs $D_{i}\left(i=1,2,3\right)$ for i,j=1,2.

Design	$D_{1}$		$D_{2}$		$D_{3}$	
Aj i#	j=1	j=2	j=1	j=2	j=1	j=2
i=1	-	8	-	8	-	8
i=2	28	0, 0, 24, 4	28	0, 0, 18, 10	28	0, 0, 12, 16

**Table 2:** Complete catalog of 27-run designs under MA, CE, and GMAF criteria.

Design	Add. Columns	WLP	Cs			AFNP and ACNP			Order
		$A_{3},\;\ldots ,A_{6}$	$C_{1}$	$C_{2}$	CC	A2 1# (C2 1#)	A2 2#	C2 2#	**G;M**
4-1.1	8	0 1	4	0	6	4	0,6	6,6	1;1
4-1.2	3	1 0	1	3	6	1,3	3,0,3	9,0,3	2;2
5-2.1	8 4	1 3 0	2	0	1	2,3	0,0,6,4	4,12,4	1;1
5-2.2	3 4	4 0 0	1	4	8	1,$0^{2}$,4	4,0,0,0,6	8,0,12	2;3
5-2.3	8 3	2 1 1	0	0	4	0,4,1	0,4,4,2	8,6,6	3;2
6-3.1	8 4 12	2 9 0 2	0	0	0	0,6	$0^{4}$,9,6	6,0,18,0,0,6	1;1
6-3.2	8 4 6	3 6 3 1	0	0	0	0,3,3	0,0,0,6,9	3,12,3,12	2;2
6-3.3	8 4 3	5 3 3 2	0	0	0	0,2,0,3,1	0,0,8,0,3,4	2,12,12,4	3;4
6-3.4	8 3 6	4 3 6 0	0	0	0	$0^{2}$,6	0,0,3,12	0,18,12	4;3
7-4.1	8 4 12 6	5 1 5 9 8	0	0	0	$0^{2}$,6,1	$0^{5}$,6,15	0,12,9,0,15,6	1;1
7-4.2	8 4 6 7	6 11 15 4	0	0	0	$0^{2}$,3,4	$0^{4}$,6,12,3	0,6,12,24	2;2
7-4.3	8 4 6 3	7 10 12 9	0	0	0	$0^{2}$,3,1,3	$0^{4}$,9,6,6	0,6,12,24	3;3
7-4.4	8 4 12 3	8 9 9 14	0	0	0	$0^{3}$,6,$0^{2}$,1	$0^{4}$,15,0,0,6	0,0,36,0,0,6	4;4
8-5.1	8 4 12 6 11	8 30 24 32	0	0	0	$0^{3}$,8	$0^{8}$,24,4	0,0,24,4,0,0,28	1;1
8-5.2	8 4 12 6 7	10 23 32 30	0	0	0	$0^{3}$,4,2,2	$0^{6}$,9,8,11	0,0,12,8,30,6	2;2
8-5.3	8 4 12 6 3	11 21 30 38	0	0	0	$0^{3}$,1,6,0,1	$0^{6}$,10,12,6	0,0,3,32,15,6	3;3
9-6.1	8 4 12 6 11 13	12 54 54 96	0	0	0	$0^{4}$,9	$0^{11}$,36	$0^{3}$,36,$0^{4}$,36	1;1
9-6.2	8 4 12 6 11 3	15 42 69 96	0	0	0	$0^{4}$,2,6,0,1	$0^{8}$,8,6,18,11	$0^{3}$,8,30,6,28	2;2
9-6.3	8 4 12 6 7 3	16 39 69 106	0	0	0	$0^{4}$,0,6,3	$0^{8}$,3,24,9	$0^{4}$,30,42	3;3
10-7.1	8 4 12 6 11 13 3	21 72 135 240	0	0	0	$0^{6}$,9,$0^{2}$,1	$0^{10}$,9,36	$0^{5}$,54,0,0,36	1;1
10-7.2	8 4 12 6 11 3 7	22 68 138 250	0	0	0	$0^{6}$,4,6	$0^{11}$,12,24,6,3	$0^{5}$,24,42,24	2;2

**Table 3:** Selected 81-run designs for 5-20 columns under MA, CE, and GMAF criteria.

Design	Add. Columns	WLP	Cs			AFNP and ACNP			Order
		$A_{3},\;\ldots ,A_{6}$	$C_{1}$	$C_{2}$	CC	A2 1# (C2 2#)	A2 2#	C2 2#	**G;M**
5-1.1	22	0 0 1	5	10	20	5	10	20	1;1
6-2.1	22 9	0 2 2 0	6	4	18	6	4,10,1	18,12	1;1
7-3.1	22 9 24	0 5 6 1	7	0	15	7	0,12,9	15,24,3	2;1
7-3.2	22 9 18	0 6 3 4	7	0	18	7	0,6,15	18,12,12	1;2
8-4.1	22 9 24 31	0 10 16 4	8	0	8	8	0,0,24,4	8,36,12	3;1
8-4.2	22 9 24 25	0 11 12 10	8	0	16	8	0,0,18,10	16,18,18,4	2;2
8-4.3	22 9 18 38	0 12 8 16	8	0	16	8	0,0,12,16	16,24,0,16	1;3
9-5.1	22 9 24 31 34	0 18 36 12	9	0	0	9	$0^{3}$,36	0,36,36	1;1
10-6.1	22 9 24 31 34 39	0 30 72 30	10	0	0	10	$0^{4}$,45	0,0,90	1;1
11-7.1	22 9 24 31	3 42 111 132	4	0	0	4,6,0,1	$0^{3}$,18	6,0,57	1;1
	34 39 3						24,13	32,15	
12-8.1	22 9 24 31 3	8 73 124 364	4	0	4	4,0,0,8	$0^{4}$,24,8	4,2,48,44	1;72
	13 6 7						0,6,12,14,2	$0^{3}$,16,18	
12-8.85	22 9 24 31 3	4 72 144 354	0	0	0	0,12	$0^{6}$,36,12	12,0,18,72	86;1
	25 13 37						12,0,6	0,12,$0^{2}$,18	
13-9.1	22 9 24 31 3	12 109 198 672	4	0	4	4,$0^{3}$,9	$0^{6}$,36,$0^{3}$	4,2,0,108	1;209
	13 6 7 12						6,0,18,18	$0^{5}$,20,22	
13-9.56	22 9 24 31 3 25	7 102 219 690	0	0	0	0,6,6,1	$0^{7}$,24,22	6,12,18,24,60,	56;1
	13 37 6						18,9,2,3	0,0,16,9,11	
14-10.1	22 9 24 31 3 25	13 147 315 1200	2	0	1	2,3,0,0,9	$0^{8}$,45,0,0	4,6,0,36,90	1;46
	13 6 7 12						6,4,27,0,9	$0^{5}$,33,0,13	
14-10.374	22 9 24 31 3 25	10 140 334 1236	0	0	0	0,0,12,2	$0^{8}$,13,24	0,24,18,0,50	375;1
	13 37 6 18						20,28,6	48,$0^{3}$,20,22	
15-11.1	22 9 24 31 3 25	14 198 486 2009	0	0	0	0,6,0,0,9	$0^{10}$,54,0	6,0,9,36,0,108	1;2
	13 37 6 7 12						0,9,33,0,0,9	$0^{5}$,36,0,0,15	
15-11.131	22 9 24 31 3 25	13 192 495 2055	0	0	0	0,0,6,9	$0^{10}$,30	0,12,33,4,0,	131;1
	13 37 6 18 7						24,8,33,9,1	84,28,$0^{4}$,36,13	
16-12.1	22 9 24 31 3 25 13	17 258 711 3275	0	0	0	0,0,6,1,9	$0^{12}$,63,0	0,12,9,36,0,0	1;2
	37 6 18 7 12						6,15,27,9	126,$0^{6}$,42,15	
16-12.531	22 9 24 31 3 25 13	16 256 720 3288	0	0	0	0,0,0,16	$0^{12}$,48	0,0,48,8,0,0	531;1
	37 6 18 7 35						16,0,48,8	112,16,$0^{5}$,56	
17-13.1	22 9 24 31 3 25 13	20 336 1014 5072	0	0	0	$0^{3}$,8,9	$0^{14}$,72,0	0,0,24,40,$0^{3}$	1;1
	37 6 18 7 35 12						0,24,40	144,$0^{7}$,64	
18-14.1	22 9 24 31 3 25 13 37	24 432 1404 7608	0	0	0	$0^{4}$,18	$0^{16}$, 81,0,	$0^{3}$,72,$0^{4}$	1;1
	6 18 7 35 12 38						0,0,72	162, $0^{8}$,72	
19-15.1	22 9 24 31 3 25 13 37	33 504 2052 10884	0	0	0	$0^{5}$,18,$0^{3}$,1	$0^{14}$,18,$0^{3}$	$0^{4}$,90,$0^{3}$,81	1;1
	6 18 7 35 12 38 15						81,0,0,72	0,99,$0^{6}$,72	
20-16.1	22 9 24 31 3 25 13 37	42 603 2808 15537	0	0	0	$0^{6}$,18,0,0,2	$0^{17}$,36,0,0,72	$0^{5}$,108,0,0,72,10	1;1
	6 18 7 35 12 38 15 16						9,54,18,$0^{3}$,1	$0^{2}$,117,$0^{4}$,54,19	

**Table 4:** Selected 243-run designs with resolution IV or higher under MA, CE, and GMAF criteria.

Design	Add. Columns	WLP	Cs			AFNP and ACNP			Order
		$A_{3},\;\ldots ,A_{6}$	$C_{1}$	$C_{2}$	CC	A2 1# (C2 1#)	A2 2#	C2 2#	**G;M**
6-1.1	63	0 0 0 1	6	15	30	6	15	30	1;1
7-2.1	63 27	0 0 3 1	7	21	42	7	21	42	1;1
8-3.1	63 27 72	0 0 8 4	8	28	56	8	28	56	1;1
9-4.1	63 27 72 79	0 0 18 12	9	36	72	9	36	72	1;1
10-5.1	63 27 72 79 93	0 0 36 30	10	45	90	10	45	90	1;1
11-6.1	63 27 72 79	0 0 66 66	11	55	110	11	55	110	1;1
	93 114								
12-7.1	63 27 72 12	0 15 72 126	12	16	60	12	16,20,24	60,54,18	1;4
	91 33 38						2,4		
12-7.2	63 27 72 79	0 14 74 110	12	14	57	12	14,26,20,6	57,66,9	6;1
	93 9 17								
13-8.1	63 27 72 79	0 25 108 201	13	8	45	13	8,17,28,	45,72,39	299;27
	93 9 17 21						23,2		
13-8.58	63 27 72 79	0 24 105 222	13	4	42	13	4,23,33	42,84,30	640;1
	9 44 57 39						17,1		
13-8.1270	63 27 45 97	0 34 75 216	13	0	60	13	0,12,24	60,24	1;1398
	9 105 20 100						24,18	36,36	
14-9.1	63 27 72 12	0 38 152 402	14	8	40	14	8,0,48	40,82,39	452;76
	91 33 38 44 50						10,24,0,1	16,5	
14-9.224	63 27 72 79	0 36 155 390	14	1	33	14	1,13,37	33,90,51,8	1387;1
	9 44 57 39 65						31,9		
14-9.2019	63 27 44 9	0 54 100 396	14	0	52	14	0,0,4,48	52,26,39	1;2019
	104 21 17 89 48						24,14,1	40,25	
15-10.1	63 27 72 12	0 62 221 576	15	2	33	15	2,7,15,20	33,38,93	601;1612
	66 44 36 30 21 20						36,19,6	36,10	
15-10.1466	63 27 72 79	0 50 231 635	15	0	15	15	0,0,40	15,100,90	1771;1
	93 9 17 44 74 117						40,25	0,5	
15-10.1778	63 27 44 9	0 72 162 640	15	0	54	15	$0^{3}$,18	54,18,45	1;1777
	104 21 17 89 33 48						72,0,15	48,45	
16-11.1	63 27 72 79 9	0 80 312 964	16	0	28	16	0,6,12,18	28,44,108	233;827
	44 57 65 21 87 109						42,24,18	20,40	
16-11.364	63 27 72 79 9	0 70 334 974	16	0	13	16	0,0,18,48	13,88	997;1
	44 57 39 65 73 21						35,15,3,1	90,44,5	
16-11.1019	63 27 44 9	0 95 252 991	16	0	60	16	$0^{4}$,60	60,0,45	1;1018
	104 21 17 89 33 39 48						45,0,15	60,75	
17-12.1	63 27 72 79 9 44	0 101 441 1531	17	0	15	17	0,0,12,24	15,72,75	210;209
	57 39 89 21 101 118						21,54,19,6	68,30,12	
17-12.274	63 27 72 79 93 9 17	0 95 450 1561	17	0	9	17	$0^{3}$,30	9,48,144	327;1
	44 74 117 21 48						59,38,9	64,0,0,7	
17-12.330	63 27 72 12 102 44	0 106 393 1660	17	0	24	17	$0^{3}$,13	24,66,66	1;312
	66 54 70 20 87 38						49,47,23,4	56,30,30	
18-13.1	63 27 72 79 9 44	0 128 606 2344	18	0	4	18	$0^{3}$,24,36	4,84,69	87;38
	57 39 89 21 113 101 118						24,54,12,3	88,30,24,7	
18-13.5	63 27 72 79 9 44 57	0 134 594 2296	18	0	20	18	$0^{3}$,18,30	20,36,84,	1;78
	54 21 87 74 109 65						24,60,18,3	92,60,0,14	
18-13.78	63 27 72 79 93 9 17	0 123 618 2352	18	0	8	18	$0^{4}$,58	8,22,144	79;1
	44 74 117 21 48 101						72,15,8	124,$0^{3}$,8	
19-14.1	63 27 72 79 93 9 17	0 168 819 3318	19	0	9	19	$0^{4}$,45	9,18,117,108	7;18
	44 21 101 113 33 39 116						36,0,72,18	45,36,0,0,9	
19-14.17	63 27 72 79 93 9 17	0 156 837 3444	19	0	9	19	$0^{5}$,108	9,0,108	15;1
	44 74 117 21 48 101 109						54,0,9	216, $0^{4}$,9	
19-14.20	63 27 72 8 52 70 56	0 180 720 3570	19	0	9	19	$0^{6}$,126	9,36,126	1;20
	38 104 54 17 36 47 85						36,9	0,90,72,0,0,9	
20-15.1	63 27 72 79 93 9 17 44	0 210 1092 4740	20	0	10	20	$0^{5}$,90,0	10,0,90,180	4;7
	21 101 113 33 39 116 118						0,90,10	0,90,$0^{3}$,10	
20-15.7	63 27 72 79 93 9 17 44	0 195 1116 4920	20	0	10	20	$0^{6}$,180	10,0,0	8;1
	74 117 21 48 101 109 113						0,0,10	360,$0^{5}$,10	
20-15.9	63 27 72 8 52 70 56	0 225 960 5100	20	0	10	20	$0^{7}$,180	10,0,180,0,0	1;9
	38 104 54 17 36 47 85 102						0,10	180,$0^{3}$,10	

## Data Availability

The original contributions presented in this study are included in the article/Supplementary Material. Further inquiries can be directed to the corresponding author(s).

## Supplementary Materials

The following supporting information can be downloaded at: https://www.mdpi.com/article/10.3390/e27070680/s1.
