# Supplementary material for: An Aliasing Measure of Factor Effects in Three-Level Regular Designs
Source: Entropy (Basel). 2025 Jun 26;27(7):680. doi: 10.3390/e27070680 (PMC12293866; doi:10.3390/e27070680)
Supplement: Supplementary file 1 [file entropy-27-00680-s001.zip › entropy-3646117-supplementary.pdf]

Supplementary Material

This section provides the MATLAB codes for calculating low-order factor aliasing.

The MATLAB codes for generating a design matrix from a saturated design.

```
function [A, F] =generateIndependentMatrix(n, m, D)
    q = n - m;
    Hq = generate_Hq(q);
    if any(D >size(Hq, 2))
        error('One or more indices in D exceed the number of columns in H_q.');
    end
    A = zeros(n, length(D));
    for i =1:length(D)
        A(1:q, i) =Hq(:, D(i));
    end
    F = zeros(n, m);
    for j =1:m
        col_idx =n - m + j;
        if col_idx <=length(D)
            F(:, j) =A(:, col_idx);
            F(col_idx, j) =F(col_idx, j) +2;
        else
            error('Confounded column index exceeds the number of columns in A');
        end
    end

end

function Hq =generate_Hq(q)
    H1 = zeros(q, 1);
    H1(1) =1;
    Hq = H1;

    for i =2:q
        [numRows, ~] =size(Hq);
        Q = zeros(numRows, 1);
        Q(i) =1;
        Hq_temp =Hq;

        Hq = [Hq_temp, Q, mod(Hq_temp +Q, 3), mod(Hq_temp +2*Q, 3)];
    end
end
```

The MATLAB codes for constructing the defining contrast subgroup matrix.

```
function G =generateEffectMatrix(F)
    [n, m] =size(F);
    Gm = F;

    uniqueCols =containers.Map();


    for i =1:m
        colStr =mat2str(F(:, i)');
        uniqueCols(colStr) = true;
    end

    for k = 2:m
```

```
        combs = nchoosek(1:m, k);
        numCombs = size(combs, 1);
        factorCombos = dec2bin(0:(2^k - 1)) - '0' + 1;

        for i = 1:numCombs
            for factorCombo = factorCombos'
                baseVec =zeros(n, 1);
                for j =1:k
                    factor =factorCombo(j);
                    baseVec =mod(baseVec +factor *F(:, combs(i, j)), 3);
                end
                if sum(baseVec ~= 0) <=4
                    colStr =mat2str(baseVec');
                    if ~isKey(uniqueCols, colStr)
                        Gm = [Gm, baseVec];
                        uniqueCols(colStr) = true;
                    end
                end
            end
        end
    end

    Gmm = [];
    for i = 1:size(Gm, 2)
        col = Gm(:, i);
        modCol = mod(2 * col, 3);
        if sum(modCol ~= 0) <= 4
            colStr = mat2str(modCol');
            if ~isKey(uniqueCols, colStr)
                Gmm = [Gmm, modCol];
                uniqueCols(colStr) =true;
            end
        end
    end

    G = [Gm, Gmm];
    validCols =[];
    for i =1:size(G, 2)
        if sum(G(:, i) ~= 0) <=4
            validCols =[validCols, G(:, i)];
        end
    end

    G = validCols;
end
```

The MATLAB codes for calculating the low-order factor aliasing values of the design.

```
function A21 =calculateA21(n, m, D)
    G = mainFunction(n, m, D);
    twoFIC =TwoFIC(n);
    k_counts =zeros(1, n +1);
    In = eye(n);


    accumulatedValue =0;
```

```
    for i =1:n
        ei = In(:, i);
        Mi =mod3HadamardProduct(ei, G);

        k = countMatchingColumns(Mi, twoFIC);
        k_counts(k +1) =k_counts(k +1) +1;

        accumulatedValue =accumulatedValue +1;

        if accumulatedValue ==n
            break;
        end
    end

    k_values =0:(find(k_counts, 1, 'last') -1);
    k_counts =k_counts(1:length(k_values));

    A21 = [k_values; k_counts];

    disp('k values:');
    disp(A21(1, :));
    disp('A21(k) values:');
    disp(A21(2, :));
end

function vectors =TwoFIC(n)
    ......(The same as above, no further elaboration)
end

function C =mod3HadamardProduct(e, G)
    if size(e, 1) ~= size(G, 1)
        error('Vector e and matrix G must have the same number of rows');
    end

    C = zeros(size(e, 1), size(G, 2));

    for j =1:size(G, 2)
        C(:, j) =mod(e +G(:, j), 3);
    end
end

function k =countMatchingColumns(M, twoFIC)
    k = 0;
    for i =1:size(M, 2)
        for j =1:size(twoFIC, 2)
            if isequal(M(:, i), twoFIC(:, j))
                k = k + 1;
                break;
            end
        end
    end
end

function G =mainFunction(n, m, D)
   ......(The same as above, no further elaboration)
end

function Hq =generate_Hq(q)
```

```
  ......(The same as above, no further elaboration)
end

function G =generateEffectMatrix(F)
 ......(The same as above, no further elaboration)
end
```

```
function A22 =calculateA22(n, m, D)
    G = mainFunction(n, m, D);
    B = generateTwoFICMatrix(n);
    B_split =splitMatrix(B, n);
    twoFIC =TwoFIC(n);
    k_counts =zeros(1, nchoosek(n, 2) +1);
    totalPairs =nchoosek(n, 2);
    accumulatedValue =0;
    for i =1:size(B_split, 2)
        Bi = B_split{i};
        Mi = mod3HadamardProduct(Bi, G);
        k = countMatchingColumns(Mi, twoFIC);
        k_counts(k +1) =k_counts(k +1) +1;
        accumulatedValue =accumulatedValue +k_counts(k +1);
        if accumulatedValue ==totalPairs
            break;
        end
    end
    k_values =0:totalPairs;
    k_counts =k_counts(1:length(k_values));
    zeroIndex =find(k_counts ==0, 1, 'last');
    k_values =k_values(1:zeroIndex);
    k_counts =k_counts(1:zeroIndex);

    A22 = [k_values; k_counts];

    disp('k values:');
    disp(A22(1, :));
    disp('A22(k) values:');
    disp(A22(2, :));
end

function vectors =TwoFIC(n)
    totalColumns =3 *nchoosek(n, 2);
    vectors =zeros(n, totalColumns);
    colIdx =1;
    for i =1:n
        for j =i+1:n
            vectors(i, colIdx) =1;
            vectors(j, colIdx) =1;
            colIdx =colIdx +1;
            vectors(i, colIdx) =1;
            vectors(j, colIdx) =2;
            colIdx =colIdx +1;
            vectors(i, colIdx) =2;
            vectors(j, colIdx) =1;
            colIdx =colIdx +1;
            vectors(i, colIdx) =2;
            vectors(j, colIdx) =2;
            colIdx =colIdx +1;
```

```
        end
    end
end

function B =generateTwoFICMatrix(n)
    totalColumns =2 *nchoosek(n, 2);
    B = zeros(n, totalColumns);
    colIdx =1;
    for i =1:n
        for j =i+1:n
            B(i, colIdx) =1;
            B(j, colIdx) =1;
            colIdx =colIdx +1;
            B(i, colIdx) =1;
            B(j, colIdx) =2;
            colIdx =colIdx +1;
        end
    end
end

function B_split =splitMatrix(B, n)
    numPairs =nchoosek(n, 2);
    B_split =cell(1, numPairs);
    for i =1:numPairs
        B_split{i} =B(:, (2*i-1):(2*i));
    end
end

function C =mod3HadamardProduct(A, B)
    [nA, m] =size(A);
    [nB, n] =size(B);
    if nA ~= nB
        error('Matrices A and B must have the same number of rows');
    end
    C = zeros(nA, m *n);
    count =1;
    for i =1:m
        for j =1:n
            C(:, count) =mod(A(:, i) +B(:, j), 3);
            count =count +1;
        end
    end
end

function k =countMatchingColumns(M, twoFIC)
    k = 0;
    for i =1:size(M, 2)
        for j =1:size(twoFIC, 2)
            if isequal(M(:, i), twoFIC(:, j))
                k = k + 1;
                break;
            end
        end
    end
end

function G =mainFunction(n, m, D)
    [A, F] =generateIndependentMatrix(n, m, D);
```

```
    G = generateEffectMatrix(F);
end

function [A, F] =generateIndependentMatrix(n, m, D)
......(The same as above, no further elaboration)
end

function Hq =generate_Hq(q)
......(The same as above, no further elaboration)
end

function G =generateEffectMatrix(F)
......(The same as above, no further elaboration)
end
```
